# Antimicrobial Potential of Scorpion-Venom-Derived Peptides

**DOI:** 10.3390/molecules29215080

**Published:** 2024-10-27

**Authors:** Zhiqiang Xia, Lixia Xie, Bing Li, Xiangyun Lv, Hongzhou Zhang, Zhijian Cao

**Affiliations:** 1 School of Biological and Food Processing Engineering, Huanghuai University, Zhumadian 463000, China; 2016202040013@whu.edu.cn (Z.X.); 20212187@huanghuai.edu.cn (L.X.); 2 Center for Evolution and Conservation Biology, Southern Marine Science and Engineering Guangdong Laboratory (Guangzhou), Guangzhou 511458, China; 3 Zhumadian Huazhong Chia Tai Co., Ltd., Zhumadian 463000, China; sinolibing@163.com (B.L.); xiangyun0309@163.com (X.L.); 4 Henan Topfond Pharmaceutical Company Limited, Zhumadian 463000, China; zhanghongzhou@topfond.com; 5 Shenzhen Research Institute, Wuhan University, Shenzhen 518057, China; 6 State Key Laboratory of Virology, College of Life Sciences, Wuhan University, Wuhan 430072, China

**Keywords:** scorpions, scorpion-venom-derived peptide, antimicrobial activity, underlying mechanism, drug development

## Abstract

The frequent and irrational use of antibiotics by humans has led to the escalating rise of antimicrobial resistance (AMR) with a high rate of morbidity-mortality worldwide, which poses a challenge to the development of effective treatments. A large number of host defense peptides from different organisms have gained interest due to their broad antibacterial spectrum, rapid action, and low target resistance, implying that these natural sources might be a new alternative to antimicrobial drugs. As important effectors of prey capture, defense against other animal attacks, and competitor deterrence, scorpion venoms have been developed as important candidate sources for modern drug development. With the rapid progress of bioanalytical and high throughput sequencing techniques, more and more scorpion-venom-derived peptides, including disulfide-bridged peptides (DBPs) and non-disulfide-bridged peptides (NDBPs), have been recently identified as having massive pharmacological activities in channelopathies, pathogen infections, and cancer treatments. In this review, we summarize the molecular diversity and corresponding structural classification of scorpion venom peptides with antibacterial, antifungal, and/or antiparasitic activity. We also aim to improve the understanding of the underlying mechanisms by which scorpion-venom-derived peptides exert these antimicrobial functions, and finally highlight their key aspects and prospects for antimicrobial therapeutic or pharmaceutical application.

## 1. Introduction

Pathogen infections are diseases related to injuries or lesions in various parts of the body caused by pathogenic microorganisms, including the skin, respiratory tract, digestive system, and urinary system, and can also cause chills, high fever, rash, joint pain, and a series of symptoms [1,2]. By now, pathogen infections have become the second leading cause of death worldwide, with *Staphylococcus aureus*, *Enterococcus faecium*, *Streptococcus pneumoniae*, *Klebsiella pneumoniae*, *Pseudomonas aeruginosa*, and Enterobacter species being the most prominent [3,4]. Since the accidental discovery of penicillin by Alexander Fleming in 1928, many other antibiotics have been discovered and used to treat various diseases caused by pathogen infections, including bacteria, fungi, and parasites. Antibiotics have saved millions of lives in the treatment of affected patients, but at the same time, bacteria have evolved resistance under applied selection pressure due to the frequent and irrational use of antibiotics by humans [5,6,7]. Hence, antibiotic resistance (AMR) in pathogenic bacteria has gradually become a new major concern in healthcare facilities where nosocomial pathogens are prevalent.

According to the World Health Organization’s (WHO) latest Global Antimicrobial Surveillance System (GLASS), antibiotic resistance is widespread in more than 3 million laboratory-confirmed pathogen infections, reported by 24,803 surveillance sites in 70 countries [8,9,10]. Most importantly, it is estimated that more than 10 million annual deaths will result from drug-resistant infections by 2050, and the global gross domestic product will be severely reduced by 1% per year from 2030 if antibiotic resistance is not curbed [11,12,13]. More than 2.8 million antibiotic-resistant cases are reported annually in the United States, resulting in more than 35,000 deaths. For example, methicillin-resistant *Staphylococcus aureus* (MRSA), an opportunistic pathogen, has been widely involved in various healthcare-acquired diseases, such as pneumonia, and skin and blood infections, and the proportion of drug-resistant bloodstream infections is 24.9% [14,15,16]. Therefore, the problem of traditional antibiotic resistance has seriously threatened human health, and it is vital to investigate more effective antimicrobial drugs from natural resources to overcome bacterial resistance.

As one of the most ancient animals on earth, scorpions have evolved over 400 million years to grow and spread widely around the world, and now more than 2800 scorpion species are distributed across 20 families as recorded on the website of The Scorpion Files (https://www.ntnu.no/ub/scorpion-files/) (accessed on 18 September 2024) [17,18,19]. In order to adapt to adversity and survive successfully, scorpions have developed a wide variety of venoms stored in the caudal gland over a long period of evolution, which can be used as a weapon for defense, competitor deterrence, or predation [20,21,22]. Among them, scorpion venom peptides are a class of small molecules composed of 20-90 amino acids, which quickly produce pain, paralysis, and even death in prey or predators by activating/blocking ion channels or receptor proteins on cell membranes, such as sodium ion channels, potassium ion channels, calcium ion channels, and chloride ion channels [18,23,24]. Natural evolutionary selection for hundreds of millions of years has endowed scorpion-venom-derived peptides with high activity and selectivity, which also makes them a potential resource library for drug development and design. In the past, the identification and functional studies of scorpion venom components were slow mainly because of their low abundance and difficulties in isolation or purification. However, thanks to the advent of omics, especially transcriptomic analysis, a large number of scorpion venom peptides have been identified, positioning the scorpion as a rich source of pharmacologically active compounds [19,25,26].

With a deep understanding of structural and functional properties, scorpion venom peptides have recently become an important drug resource for the diagnosis and treatment of related diseases, especially in antibacterial, antifungal, and antiparasitic activity [24,27,28,29]. However, most non-disulfide-bridged peptides (NDBPs), a novel class of scorpion-venom-derived peptides with no specific channel targets, showed obvious antimicrobial activity only in vitro, and their comprehensive antimicrobial targets and mechanisms still need to be further elucidated, as well as the optimization of drug delivery in vivo. Therefore, the structural classification and mechanism elucidation of these multifunctional scorpion-venom-derived peptides will provide an important breakthrough in the utilization of scorpion resources in the future, positioning them as new-generation candidates to develop novel and high-quality therapeutic pharmaceuticals. In this review, we mainly focus on the molecular diversity and typical structural characteristics of scorpion venom peptides with antimicrobial activity. We also aim to establish the potential relationship between the molecular characteristics and functional applications of scorpion venom peptides to provide a research basis for the drug development and clinical utilization of scorpion resources.

## 2. Composition of Scorpion Venoms

In order to achieve the goal of efficient predation and defense against natural enemies, scorpion venoms have produced amazing molecular diversity through a long natural evolution process, showing high activity, high specificity, structural diversity, and occurring in large quantities. In addition to lipids, nucleotides, free amino acids, and other non-protein components, scorpion venom peptides and some proteins are the main active components, including neurotoxins, scorpine-like peptides, long cationic antimicrobial peptides, serine proteases, phospholipases, salivary proteins, and so on [29,30]. Based on the structural and functional properties, scorpion venom peptides can be classified into two main groups: disulfide-bridged peptides (DBPs) and non-disulfide-bridged peptides (NDBPs) [31]. Typically, most scorpion DBPs contain three or four disulfide bridges structurally composed of 13 to 70 amino acids and are also classified by targeted channel names due to their ability to significantly alter the permeability of certain ion channels, mainly including sodium channel toxins (NaTx), potassium channel toxins (KTx), calcium channel toxins (CaTx), chloride channel bound toxins (ClTx), and TRP channel toxins (TRPTx) [32,33,34,35,36]. Furthermore, a large number of scorpion neurotoxins displaying conserved structure–function relationships have been extensively studied, and increasingly provide important leads and candidates for pharmaceutical research in channelopathies, such as analgesic, antiepileptic, and autoimmune activities [37]. Unlike DBPs, the scorpion NDBPs without disulfide bridges are often composed of 13–56 amino acids and have attracted increasing interest in recent decades due to their diverse biological functions and potential pharmacological applications, such as antibacterial, antifungal, and antiparasitic effects [38,39,40]. Accordingly, NDBPs can be divided into two categories based solely on the amino acid length of the peptide: short-chain NDBPs with 10–25 amino acids and long-chain NDBPs with approximately 50 amino acids. Generally, the C-terminal of mature short-chain NDBPs has strong cation characteristics, such as lysine, arginine, and histidine, which allows them to easily bind to bacterial cell membranes and perform strong antimicrobial activity against bacteria, viruses, or fungi.

## 3. Antimicrobial Potential of Scorpion Venom Peptides

**Figure 1:**
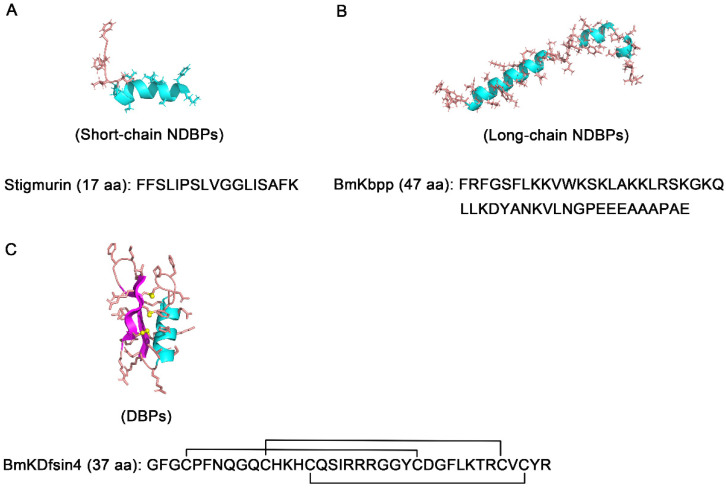
The primary sequences and 3D structures of representative scorpion venom peptides with antibacterial activity. (**A**) The sequence and 3D structure of stigmurin classified into the short-chain NDBPs group (PDB code: 6VL2) [44]. (**B**) The sequence and 3D structure of BmKbpp classified into the long-chain NDBPs group was modeled using the SWISS-MODEL server based on the parabutoporin template (AlphaFold Protein Structure Database code: AF-P83312-F1-v4). (**C**) The sequence and 3D structure of BmKDfsin4 classified into the DBPs group was modeled using the SWISS-MODEL server based on the BmKDfsin3 template (PDB code: 5XA6).

Considering the advantages of broad-spectrum activity, rapid mechanisms of action, and low drug resistance, the use of antimicrobial peptides (AMPs) against invading pathogens has proven to be a promising strategy for developing new generations of antibiotics. Because the physical process of venom injection may damage the caudal ganglia, resulting in further pathogen infection, scorpions are expected to possess an antimicrobial response to protect themselves from the invasion of microbes through unique mechanisms [41,42,43]. With the structural and functional characterization of scorpion venom components, a significant percentage of scorpion-venom-derived peptides with antimicrobial properties have been identified, such as those targeting bacteria, fungi, and parasites. Depending on their sequence length and disulfide bridges, scorpion-venom-derived antimicrobial peptides can be classified into three major groups: short-chain NDBPs, long-chain NDBPs, and DBPs, which are respectively represented by stigmurin, BmKbpp, and BmKDfsin4 (Figure 1). Although NDBPs have no specific channel targets, their unique structure and multifunctional biological activities will expand the abundance and potential for pharmacological applications of scorpion resources, particularly in bacterial, fungal, and parasitic infections. Together, these peptides are positioned as potential therapeutic candidates for the design and development of new-generation antibacterial drugs.

### 3.1. Antibacterial Peptides Derived from Scorpion Venoms

The increased use of antibiotics in recent years to treat bacterial infections has resulted in the evolution of antimicrobial resistance, and approximately 700,000 deaths are attributed annually to antimicrobial-resistant bacteria, primarily to *Acinetobacter baumanella*, *Klebsiella pneumoniae*, and *Staphylococcus aureus* [45,46]. This poses serious threats to human health and leads to a significant economic burden on national health security. To date, various broad-spectrum antibacterial peptides with activity against a diverse group of Gram-positive and Gram-negative bacteria have been identified in scorpion venoms, presenting them as candidate antibacterial drugs. Among them, more than forty-seven short-chain and nine long-chain NDBPs with antibacterial properties have been isolated from different scorpion species, including *Mesobuthus martensii*, *Opisthacanthus madagascarieni*, *Tityus stigmurus*, *Lychas mucronatus*, *Androctonus amoreuxi*, *Vaejovis punctatus*, *Pandinus imperator*, *Androctonus aeneas*, and *Heterometrus spinifer*, suggesting that short-chain NDBPs with simple structures and short sequences can be effectively used as antibacterial drug candidates (Table 1). Furthermore, thirty short-chain NDBPs showed broad-spectrum activity against both Gram-positive and Gram-negative bacteria, whereas the remaining peptides, except Hp1404, were more specific against a diverse group of Gram-positive bacteria [47]. For instance, our group previously demonstrated that both BmKn2 and Kn2-7, isolated from the scorpion *Mesobuthus martensii*, were composed of 13 amino acids without disulfide bridges and shared antibacterial activity against both Gram-positive and Gram-negative bacteria, whereas Kn2-7 designed from BmKn2 showed increased inhibitory activity to clinical antibiotic-resistant strains because of its shorter sequence, indicating that the peptide Kn2-7 can be developed as a potential antibacterial agent [48,49]. Moreover, stigmurin, derived from the scorpion *Tityus stigmurus*, is composed of 17 amino acids without disulfide bridges and exhibits antibacterial activity against Gram-positive bacteria [50]. Because of the higher positive net charge and hydrophobicity induced by amino acid substitutions, the four analog peptides StigA6, StigA16, StigA25, and StigA31 not only showed an antibacterial effect superior to that of stigmurin but also improved the antibacterial spectrum (both Gram-positive and Gram-negative bacteria). Thus, scorpion-venom-derived peptides with antibacterial activity can be effectively used as scaffolds to design more promising candidates [51,52].

Besides stigmurin, certain scorpion venom peptides specifically exhibiting extensive antibacterial properties against Gram-positive bacteria have been demonstrated, such as Pantinin-1, Pantinin-2, Pantinin-3, StCT2, TsAP-2, Marcin-18, AaeAP1, and AaeAP2 [53,54,55,56,57]. Two novel antimicrobial peptides, AaeAP1 and AaeAP2, isolated from the North African scorpion *Androctonus aeneas*, contain 17 amino acids without disulfide bridges. Both exhibited more selective growth-inhibitory activities against *Staphylococcus aureus* (16 mg/L) than against *Escherichia coli* (512 mg/L), indicating that they are promising candidates for the treatment of clinical antibiotic-resistant strains, especially Gram-positive bacteria [56].

**Table 1:** Short-chain NDBPs derived from scorpion venoms with antibacterial activity.

Scorpion Species	Peptides	Amino Acid Sequence and Length	* Hydrophobicity (kcal × mol^−1^)	* Molecular Weight (Da)	* pI	* Net Charge	Antimicrobial Activity	References
*M. martensii*	BmKn2	FIGAIANLLSKIF (13)	4.88	1405.8	9.93	+1	Gram-positive and Gram-negative bacteria	[48]
*M. martensii*	Kn2-7	FIGAIAKLLKKIF (13)	9.17	1460.9	10.86	+3	Gram-positive and Gram-negative bacteria	[49]
*O. Madagascariensis*	IsCT1	ILGKIWEGIKSLF (13)	10.23	1502.8	9.74	+1	Gram-positive and Gram-negative bacteria	[58]
*O. Madagascariensis*	IsCT2	IFGAIWNGIKSLF (13)	4.69	1464.8	9.93	+1	Gram-positive and Gram-negative bacteria	[58]
*V. subcristatus*	VsCT1	FLKGIIDTVSNWL (13)	8.05	1504.8	6.71	0	Gram-positive and Gram-negative bacteria	[59]
*V. subcristatus*	VsCT2	FLKGIIDTVSKLF (13)	10.38	1479.8	9.74	+1	Gram-positive and Gram-negative bacteria	[59]
*V. mexicanus*	VmCT1	FLGALWNVAKSVF (13)	5.23	1450.8	9.93	+1	Gram-positive and Gram-negative bacteria	[60]
*V. mexicanus*	VmCT2	FLSTLWNAAKSIF (13)	4.59	1496.8	9.93	+1	Gram-positive and Gram-negative bacteria	[60]
*U. yaschenkoi*	UyCT3	ILSAIWSGIKSLF (13)	4.07	1433.8	9.93	+1	Gram-positive and Gram-negative bacteria	[61]
*U. yaschenkoi*	UyCT5	IWSAIWSGIKGLL (13)	4.38	1442.8	10.14	+1	Gram-positive and Gram-negative bacteria	[61]
*U. yaschenkoi*	Uy17	ILSAIWSGIKGLL (13)	5.22	1369.8	10.14	+1	Gram-positive and Gram-negative bacteria	[61]
*U. yaschenkoi*	Uy192	FLSTIWNGIKGLL (13)	4.77	1460.8	10.14	+1	Gram-positive and Gram-negative bacteria	[61]
*U. manicatus*	Um4	FFSALLSGIKSLF (13)	3.73	1428.8	9.93	+1	Gram-positive and Gram-negative bacteria	[61]
*U. manicatus*	Um5	IFKAIWSGIKSLF (13)	5.95	1508.9	10.59	+2	Gram-positive and Gram-negative bacteria	[61]
*U. yaschenkoi*	UyCT1	GFWGKLWEGVKNAI (14)	13.21	1603.8	9.94	+1	Gram-positive and Gram-negative bacteria	[61]
*U. manicatus*	Um3	GFWGKLWEGVKSAI (14)	12.82	1576.6	9.94	+1	Gram-positive and Gram-negative bacteria	[61]
*T. stigmurus*	StigA6	FFSLIPKLVKGLISAFK (17)	7.43	1907.1	10.86	+3	Gram-positive and Gram-negative bacteria	[51]
*T. stigmurus*	StigA16	FFKLIPKLVKGLISAFK (17)	9.77	1948.2	11.03	+4	Gram-positive and Gram-negative bacteria	[51]
*T. stigmurus*	StigA25	FFSLIPSLVKKLIKAFK (17)	9.08	1978.2	11.03	+4	Gram-positive and Gram-negative bacteria	[52]
*T. stigmurus*	StigA31	FFKLIPKLVKKLIKAFK (17)	13.76	2060.3	11.25	+6	Gram-positive and Gram-negative bacteria	[52]
*L. mucronatus*	Mucroporin	LFGLIPSLIGGLVSAFK (17)	4.59	1731.0	9.8	+1	Gram-positive and Gram-negative bacteria	[62]
*L. mucronatus*	mucroporin-M1	LFRLIKSLIKRLVSAFK (17)	10.22	2031.3	12.51	+5	Gram-positive and Gram-negative bacteria	[62]
*U. yaschenkoi*	Uy234	FPFLLSLIPSAISAIKRL (18)	3.39	1985.2	11.55	+2	Gram-positive and Gram-negative bacteria	[61]
*A. amoreuxi*	AamAP1	FLFSLIPHAIGGLISAFK (18)	5.15	1930.1	9.80	+1	Gram-positive and Gram-negative bacteria	[63]
*A. amoreuxi*	AamAP2	FPFSLIPHAIGGLISAIK (18)	7.13	1880.1	9.80	+1	Gram-positive and Gram-negative bacteria	[63]
*U. manicatus*	Um2	ISQSDAILSAIWSGIKSLF (19)	8.78	2035.1	6.55	0	Gram-positive and Gram-negative bacteria	[61]
*V. punctatus*	VpAmp1.0	LPFFLLSLIPSAISAIKKI (19)	3.26	2070.3	10.65	+2	Gram-positive and Gram-negative bacteria	[64]
*V. punctatus*	VpAmp2.0	FWGFLGKLAMKAVPSLIGGNKSSSK (25)	16.02	2622.4	11.03	+4	Gram-positive and Gram-negative bacteria	[64]
*C. suffuses*	Css54	FFGSLLSLGSKLLPSVFKLFQRKKE (25)	14.79	2868.6	11.02	+4	Gram-positive and Gram-negative bacteria	[65]
*H. spinifer*	HsAp	SGTSEKERESGRLLGVVKRLIVCFRSPFP (29)	27.76	3246.7	10.83	+3	Gram-positive and Gram-negative bacteria	[66]
*P. imperator*	Pantinin-2	IFGAIWKGISSLL (13)	4.76	1403.8	10.14	+1	Gram-positive bacteria	[53]
*P. imperator*	Pantinin-3	FLSTIWNGIKSLL (13)	4.08	1490.8	10.14	+1	Gram-positive bacteria	[53]
*P. imperator*	Pantinin-1	GILGKLWEGFKSIV (14)	12.04	1545.9	9.93	+1	Gram-positive bacteria	[53]
*H. petersii*	Hp1470	IFKAIWSGINRLF (13)	5.35	1563.9	11.53	+2	Gram-positive bacteria	[67]
*S. tibetanus*	StCT2	GFWGKLWEGVKSAI (14)	12.82	1576.8	9.94	+1	Gram-positive bacteria	[54]
*T. stigmurus*	Stigmurin	FFSLIPSLVGGLISAFK (17)	3.44	1795.0	9.80	+1	Gram-positive bacteria	[50]
*I. maculates*	Imcroporin	FFSLLPSLIGGLVSAIK (17)	3.90	1761.0	9.80	+1	Gram-positive bacteria	[68]
*T. serrulatus*	TsAP-2	FLGMIPGLIGGLISAFK (17)	5.20	1732.9	9.80	+1	Gram-positive bacteria	[55]
*M. martensii*	Marcin-18	FFGHLFKLATKIIPSLFR (18)	7.31	2134.2	11.68	+3	Gram-positive bacteria	[57]
*U. yaschenkoi*	Uy234	FPFLLSLIPSAISAIKRL (18)	3.39	1985.2	11.55	+2	Gram-positive bacteria	[69]
*O. glabrifrons*	Opisin	FWSWLMKAATKLLPSMLGS (19)	5.19	2166.1	10.58	+2	Gram-positive bacteria	[70]
*A. aeneas*	AaeAP1	FLFSLIPSVIAGLVSAIRN (19)	2.78	2016.2	10.60	+1	Gram-positive bacteria	[56]
*A. aeneas*	AaeAP2	FLFSLIPSAIAGLVSAIRN (19)	3.74	1988.1	10.60	+1	Gram-positive bacteria	[56]
*C. tricostatus*	Ctriporin	FLWGLIPGAISAVTSLIKK (19)	6.74	2013.2	10.57	+2	Gram-positive bacteria	[71]
*P. imperator*	Pandinin-2	FWGALAKGALKLIPSLFSSFSKKD (24)	15.18	2610.4	10.62	+3	Gram-positive bacteria	[72]
*H. spinifer*	Heterin-2	FWGALAKGALKLIPSLVSSFTKKD (24)	16.22	2576.4	10.62	+3	Gram-positive bacteria	[73]
*H. petersii*	Hp1404	GILGKLWEGVKSIF (14)	12.04	1545.9	9.74	+1	Gram-negative bacteria	[47]

* PeP Draw: www.tulane.edu/~biochem/WW/PepDraw/index.html, accessed on 22 October 2024.

Unlike short-chain NDBPs, a new class of antibacterial peptides ranging from 41 to 49 amino acids in length has been characterized as long-chain NDBPs (Table 2), which are more effective against Gram-negative bacteria than against Gram-positive ones, such as hadrurin, vejovine, BmKbpp, or meucin-49 [74,75,76,77]. For instance, hadrurin, discovered in the Mexican scorpion *Hadrurus aztecus*, contains 41 amino acids in sequence without disulfide bridges and has been found to display potent antibacterial activity at low micromolar concentrations against Gram-negative bacteria, including *Salmonella typhi*, *Klebsiella pneumoniae*, *Enterococcus cloacae*, *Pseudomonas aeruginosa*, *Escherichia coli*, and *Serratia marscences* [75]. Recently, a multifunctional scorpion venom peptide, meucin-49, containing 49 amino acids without disulfide bridges, was isolated from the scorpion *Mesobuthus eupeus* and exhibited highly potent inhibitory activity against both Gram-negative and Gram-positive bacteria, mainly by destroying the bacterial cell membrane and causing leakage of intracellular components [74].

Besides NDBPs with antibacterial activity, certain scorpion-venom-derived peptides with three disulfide bridges, also known as β-KTx or scorpine-like peptides due to their two different structural domains, have been shown to possess broad-spectrum antibacterial activity [81] (Table 3). Notably, scorpine, isolated from the scorpion *Pandinus imperator*, is composed of 75 amino acids with a molecular mass of 8350 Da and has been shown to have a wide variety of profiles against both Gram-positive and Gram-negative bacteria due to its hybrid similarity with some defensins, including *Enterococcus faecalis*, *Bacillus subtilis*, *Klebsiella pneumoniae*, *Escherichia coli*, and *Staphylococcus aureus* [82]. LaIT3, a novel insecticidal peptide isolated from the *Liocheles australasiae* venom, belongs to the scorpine-like peptides consisting of two structural domains: an N-terminal α-helical domain and a C-terminal cystine-stabilized domain. It showed significant antibacterial activity against *Escherichia coli*. Although the discovery of antibacterial scorpion peptides is varied and abundant, the main limitation of their therapeutic application is that their minimum inhibitory concentration MIC values against bacteria are very close to the concentration at which they produce cytotoxicity in mammalian cells [83]. Therefore, effectively improving bactericidal selectivity must be a primary consideration for the development of new antibacterial peptides in the future.

### 3.2. Antifungal Peptides Derived from Scorpion Venoms

Invasive fungal infections are diseases in which pathogenic fungi invade the body and multiply in the skin, viscera, blood, or central nervous system, causing further tissue damage, inflammatory responses, and organ dysfunction. Owing to their strong invasiveness and high morbidity, it has been reported that the prevalence of invasive fungal infections has increased by at least 150 million cases every year. These infections cause a series of severe clinical symptoms in patients hospitalized in intensive care units or infected with the human immunodeficiency virus (HIV), as well as in organ or cell transplant recipients, posing significant concerns for both clinicians and researchers [87,88,89]. As the most important opportunistic fungal pathogens, *Candida* species, including *Candida albicans*, *Candida parapsilosis*, *Candida krusei*, *Candida glabrata*, and *Candida tropicalis*, are the most common causes of fungal infections worldwide, presenting significant challenges to many medical centers in addition to *Aspergillus* and *Cryptococcus* [90]. Although several drugs, such as azoles, polyenes, and echinocandins, have been approved for the treatment of *Candida* infections, the discovery of new antifungal candidates for invasive fungal infections is crucial because of the poor clinical efficacy and drug resistance challenges associated with these existing drugs [91].

During the search for antibacterial scorpion venom peptides, several peptides showed inhibitory activity against fungi, especially *Candida* spp. To date, more than twenty-seven scorpion-venom-derived peptides have been confirmed to exert significant antifungal activity, and twenty-five peptides without disulfide bridges have been characterized in the NDBP family (Table 4). Based on the difference in their amino acid lengths, twenty-three peptides isolated from various scorpions, such as *Tityus stigmurus*, *Tityus obscurus*, *Tityus serrulatus*, *Androctonus amoreuxi*, and *Androctonus aeneas*, were classified as short-chain NDBPs, except for serrulin, opistoporin 1, and parabutoporin, which have longer amino acid sequences [80,92]. Because *Candida* plays a significant role as the most frequent model organism used in basic research assays, the antifungal effects of most scorpion venom peptides were evaluated by testing the growth inhibition of *Candida* spp. For example, six peptides discovered from the scorpion *Tityus stigmurus* have been demonstrated to have potent antifungal activity at low micromolar concentrations against *Candida albicans*, *Candida krusei*, and *Candida glabrata*, and four analog peptides (StigA6, StigA16, StigA25, and StigA31) derived from stigmurin displayed superior antifungal effects compared to the native peptide, suggesting that peptides from this species can be considered as scaffolds to design more promising candidates for the treatment of candidiasis [50,51,52,93]. Similarly, ToAP1, ToAP2, and ToAP3, identified from the scorpion *Tityus obscurus*, are short-chain NDBPs with 17 or 26 amino acids, and they have exhibited potent activity against *Candida* spp. and *Cryptococcus neoformans*, with MIC values ranging from 3.12 to 200 μM, providing potential therapeutic applications against a wide range of fungi [94]. In addition to short-chain NDBPs with antifungal activity, other categories of scorpion venom peptides also possess antifungal properties, including long-chain NDBPs, KTx, and NaTx. For instance, the long-chain NDBPs opistoporin 1, isolated from the scorpion *Opistophtalmus carinatus*, and parabutoporin, discovered in the scorpion *Parabuthus schlechteri*, differ only by one amino acid, and both peptides displayed a 50% growth inhibitory effect at a dose of 2 μM on *Saccharomyces cerevisiae* via membrane permeabilization [80].

Recently, a multifunctional toxin Ts1, composed of 61 amino acids with four disulfide bridges, has been isolated from the scorpion *Tityus serrulatus* and exhibited 100% inhibition against *Aspergillus nidulans* at a concentration of 4.36 μM [97]. This finding suggests that Ts1, as the first cysteine-containing scorpion toxin, could be used as a template in the search for new mechanisms of action of antifungal drugs (Table 5). Moreover, the neurotoxin Ts8, containing 60 amino acids with three disulfide bridges, was also identified from the scorpion *Tityus serrulatus* and has been demonstrated to inhibit the growth of *Pichia pastoris* and Kv1.3 channel activity, indicating that the antimicrobial mechanism of Ts8 is closely related to its channel blocking capability, similar to other scorpine-like peptides [98]. Overall, these scorpion-venom-derived peptides provide a promising template for the design of engineered scorpion AMPs for future therapeutic applications.

### 3.3. Antiparasitic Peptides Derived from Scorpion Venoms

Parasitic diseases are infectious diseases caused by various parasites that can live in or on other organisms, such as *Trypanosoma cruzi*, *Plasmodium berghei*, and *Taenia crassiceps*, and are most prevalent in developing countries, especially in tropical and subtropical regions. Annually, more than two billion people worldwide are affected by parasitic diseases, resulting in high levels of morbidity and mortality with significant impacts on both human health and livestock production [99]. Although antiparasitic drugs are the primary management strategy for parasitic diseases, they commonly exhibit drug resistance and severe side effects; therefore, it is crucial to discover and develop novel compounds for antiparasitic therapy [100]. Animal venoms have shown unique potential as natural sources of active molecules in many fields of pathogen prevention. Scorpion venom peptides derived from multiple scorpion species such as *Tityus stigmurus*, *Hoffmannihadrurus gertschi*, *Mesobuthus eupeus*, and *Pandinus imperator* have been shown to possess inhibitory effects against different parasites, and over nine peptides with antiparasitic activity have been widely reported [41]. Most of these antiparasitic peptides belong to short-chain NDBPs (Table 6), and only two peptides from the scorpions *Hoffmannihadrurus gertschi* and *Pandinus imperator* were classified into the KTx group (Table 7), suggesting that the structure and classification of antiparasitic peptides are relatively conserved.

Stigmurin, isolated from the scorpion *Tityus stigmurus*, contains 17 amino acids and lacks a disulfide bridge. It demonstrates strong inhibitory activity in antibacterial and antifungal fields but not antiparasitic activity [50]. Interestingly, four peptide analogs of stigmurin, named StigA6, StigA16, StigA25, and StigA31, not only showed antibacterial and antifungal effects superior to those of the native peptide but also efficiently inhibited the growth of epimastigote forms of *Trypanosoma cruzi* [51,52]. Meanwhile, StigA6 and StigA16 induced 100% parasite death after 12 h of incubation at concentrations of 10 and 25 µM respectively, indicating that both have the potential to be used as therapeutic agents in antiparasitic drugs. In addition to the above four short-chain NDBPs, meucin-24, containing 24 amino acids, and meucin-25, containing 25 amino acids, were identified from the scorpion *Mesobuthus eupeus* and shared significant antimalarial activities with intraerythrocytic *Plasmodium falciparum* without harming mammalian cells, making them candidates for the discovery of potential antimalarial drugs [101].

**Table 6:** NDBPs derived from scorpion venoms with antiparasitic activity.

Scorpion Species	Peptides	Amino Acid Sequence and Length	* Hydrophobicity (kcal × mol^−1^)	* Molecular Weight (Da)	* pI	* Net Charge	Antimicrobial Activity	References
*V. mexicanus*	VmCT1	FLGALWNVAKSVF (13)	5.23	1450.8	9.93	+1	*T. cruzi*	[102]
*T. stigmurus*	StigA6	FFSLIPKLVKGLISAFK (17)	7.43	1907.1	10.86	+3	*T. cruzi*	[51]
*T. stigmurus*	StigA16	FFKLIPKLVKGLISAFK (17)	9.77	1948.2	11.03	+4	*T. cruzi*	[51]
*T. stigmurus*	StigA25	FFSLIPSLVKKLIKAFK (17)	9.08	1978.2	11.03	+4	*T. cruzi*	[52]
*T. stigmurus*	StigA31	FFKLIPKLVKKLIKAFK (17)	13.76	2060.3	11.25	+6	*T. cruzi*	[52]
*M. eupeus*	Meucin-24	GRGREFMSNLKEKLSGVKEKMKNS (24)	36.93	2752.4	10.72	+4	*P. falciparum*	[101]
*M. eupeus*	Meucin-25	VKLIQIRIWIQYVTVLQMFSMKTKQ (25)	7.94	3093.7	10.92	+4	*P. falciparum*	[101]

* PeP Draw: www.tulane.edu/~biochem/WW/PepDraw/index.html, accessed on 22 October 2024.

**Table 7:** DBPs derived from scorpion venoms with antiparasitic activity.

Scorpion Species	Peptides	Peptide Length (S–S Bridge)	Sequence	Classification	Antimicrobial Activity	References
*H. gertschi*	Hge36	48 (3)	VHKMAKNQFGCFANVDVKGDCKRHCKAEDKEGICHGTKCKCGVPISYL	KTx	*T. crassiceps*	[103]
*P. imperator*	Scorpine	75 (3)	GWINEEKIQKKIDERMGNTVLGGMAKAIVHKMAKNEFQCMANMDMLGNCEKHCQTSGEKGYCHGTKCKCGTPLSY	KTx	*P. berghei*	[82]

In addition to antiparasitic peptides classified as short-chain NDBPs, the novel peptide scorpine and a scorpine-like peptide Hge36, isolated from the scorpion *Pandinus imperator* and *Hoffmannihadrurus gertschi*, respectively, have been shown to display potassium-channel-blocking activities as well as potent inhibitory effects on parasites [82,103]. In contrast, scorpine, composed of 75 amino acids with three disulfide bridges, mainly inhibited *Plasmodium berghei* development, while Hge36, which contains 48 amino acids with three disulfide bridges, primarily reduced the viability of *Taenia crassiceps* and apoptosis in the trophozoites of *Entamoeba histolytica* in vitro experiments. Therefore, these scorpion-venom-derived peptides have potential as therapeutic agents for parasitic diseases, particularly human cysticercosis.

## 4. Discussion

### 4.1. Trends in Various Targets and Mechanisms of Antimicrobial Peptides

**Figure 2:**
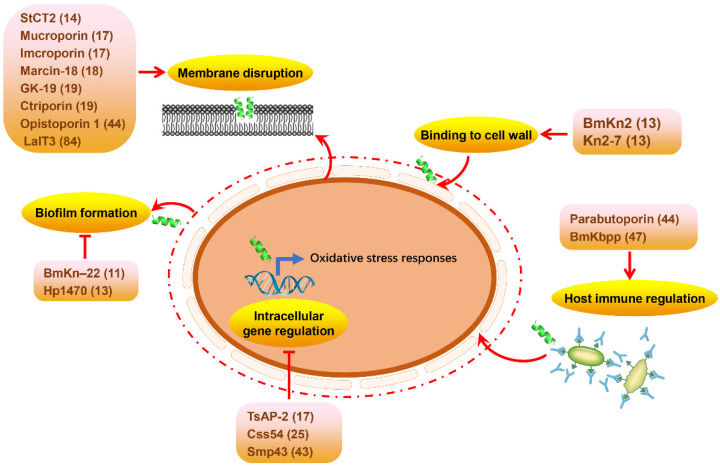
Schematic of the main targets of scorpion-venom-derived antimicrobial peptides. In the model, the numbers in parentheses represent the lengths of amino acid sequences.

Massive scorpion-venom-derived peptides have been shown to have a broad spectrum of activity against bacteria, fungi, and parasites, making them potential therapeutic candidates for the design and development of new-generation antimicrobial drugs. However, a significant percentage of them have not been identified with a clear target and molecular mechanism. Although the antimicrobial targets of some scorpion venom peptides have been revealed to be closely related to membrane disruption, binding to cell wall components, inhibiting biofilm formation, and regulating intracellular pathways and host immune responses (Figure 2), the underlying molecular mechanism remains to be further elucidated [104]. Importantly, disrupting the microbial cell membrane is the main mechanism of scorpion-venom-derived antimicrobial peptides due to electrostatic forces between their positive amino acid residues and the negative charges exposed at the pathogen surfaces, such as StCT2, mucroporin, imcroporin, marcin-18, GK-19, ctriporin, opistoporin 1, and LaIT3 [54,57,80,85]. In addition, the components of the cell wall can also be the main molecular targets for scorpion-venom-derived antimicrobial peptides, such as lipopolysaccharides (LPS) found exclusively in Gram-negative bacteria, and lipoteichoic acid (LTA), which is specific to Gram-positive bacteria. For example, Kn2-7, an analog peptide from BmKn2, a native peptide from the scorpion Mesobuthus martensii, promoted the disruption of Staphylococcus aureus and Escherichia coli cell walls through binding to LTA and LPS, respectively [49]. Interestingly, BmKn–22, a modified peptide of the parental BmKn–2 scorpion venom peptide, and Hp1470, identified from the venom of the scorpion Heterometrus petersii, only inhibited the biofilm formation but did not kill bacteria, which provides a new direction for developing antibacterial agents with different modes of action from natural scorpion venoms [67,105]. Recently, regulating intracellular pathways and host immune responses have also been described as strategies to exert antimicrobial activity for scorpion venom peptides. For example, Smp43 from the Egyptian scorpion Scorpio mauruspalmatus not only disrupts bacterial cell membranes but can also interfere with intracellular gene expression, and BmKbpp, a novel cationic and α-helical peptide from the Chinese scorpion Mesobuthus martensii, can act as a signaling molecule involving innate immune regulation at low concentrations [77,106]. Together, the structural variations between scorpion-venom-derived antimicrobial peptides define their distinct action targets, resulting in a diverse antibacterial spectrum. More research is needed to explain the antimicrobial mechanism in the future so that antimicrobial peptides can be used specifically to treat different clinical diseases.

### 4.2. Trends in Modifying the Stability and Bioavailability of Antimicrobial Peptides

Antimicrobial resistance triggered by inappropriate or excessive prescription of antibiotics or through overuse in agriculture is clearly an emerging public health problem, and the discovery of new substances is mandatory to fight against it [107]. Despite the promising research and development of peptide drugs in recent years, there are still inherent disadvantages, such as poor in vivo stability leading to a short half-life and the need for frequent administration by patients. Enhancing peptide stability and bioavailability has become a common issue that peptide drugs urgently need to address. As a novel class of antimicrobial peptides derived from scorpion venoms, defensins are cysteine-rich cationic peptides with cystine-stabilized α/β motifs. They have defensive functions against a broad array of infectious pathogens like bacteria, fungi, parasites, and viruses, providing potential candidate molecules for the treatment of antibiotic-resistant pathogens. Based on the draft-genome sequence of the scorpion *Mesobuthus martensii* (Scorpions: Buthidae), our group previously annotated and confirmed six defensin genes consisting of 37 or 38 mature peptide residues and named them BmKDfsin1, BmKDfsin2, BmKDfsin3, BmKDfsin4, BmKDfsin5, and BmKDfsin6, respectively [108]. However, due to the poor structural stability and high toxicity of the other five peptides, only the antibacterial activity of BmKDfsin4 has been reported [84]. Additionally, numerous scorpion-venom-derived peptides with antimicrobial activity have been identified in vitro, such as AaeAP1, IsCT, Pantinin-2, AaeAP1, and their analogs, and exactly how bioavailable and bactericidal they are in vivo is still unclear. Therefore, improved bioavailability and stability of scorpion-venom-derived peptides will expand the abundance and potential of scorpion resources for pharmacological applications in the future.

### 4.3. Trends in Structure Modification and Grafting of Antimicrobial Peptides

To tackle the problem of multi-resistance caused by different pathogenic infections and the irrational use of antibiotics, a number of antimicrobial peptides derived from scorpion venoms have been found and developed recently [31,109]. However, the MIC values of most naturally active antimicrobial peptides extracted from animals are at the micromolar (µM) level, and none of them are currently being developed for clinical purposes, as the concepts involving their structural organization, toxicity, stability, and solubility remain ambiguous. Therefore, it is necessary to increase their activity in vivo through modification of their primary sequence and optimization of their secondary structure before future pharmacological application. Amorim-Carmo et al. have noted that some physicochemical properties can be considered when developing an AMP derived from scorpion venoms, including peptide sequence and conformation, amphipathicity, hydrophobicity, hydrophobic moment, C-terminal amidation, net charge, polar angle, and hydrophobic angle [104]. For example, BmKTX, comprising 37 amino acids, was identified from *M. martensii* and can inhibit Kv1.3 channels with a 50% inhibitory concentration (IC_50_) of 0.2 nM, rendering it a potential drug for treating autoimmune diseases. The modified analogs BmKTX-D33H, tailored by replacing the Asp33 residue in BmKTX with His, and ADWX-1, designed by replacing the Gly11 and Ile28 residues in BmKTX-D33H with Arg and Thr, respectively, exhibit approximately 10,000- and 340-fold greater selectivity for the Kv1.3 channel, respectively, validating their efficacy as autoimmune disease therapeutics [110,111]. Therefore, with a thorough understanding of their structural and functional properties, more novel molecules with high activity and selectivity based on the natural framework of scorpion venom peptides will provide a potential resource for developing novel and high-quality therapeutic agents.

## 5. Conclusions

In conclusion, this review mainly focused on the molecular diversity and typical structural characteristics of scorpion venom peptides with antimicrobial activity and aimed to establish the potential relationship between their molecular characteristics and functional applications. Although a rich and diverse group of scorpion venom peptides showing potent therapeutic applications against pathogen infections has been reported recently, comprehensive evaluations of their antimicrobial targets and mechanisms, as well as the optimization of drug delivery in vivo, remain to be elucidated. Therefore, it is still necessary to further elucidate their antimicrobial molecular mechanism and improve their antimicrobial activity before validating their potential as candidates for the development of novel, high-quality therapeutic pharmaceuticals. In the future, these highly active and selective antimicrobial peptides derived from diverse scorpions will provide a solution for the replacement of antibiotics and represent a good starting point for the drug development and clinical utilization of scorpion resources.

## Figures and Tables

**Table 2:** Long-chain NDBPs derived from scorpion venoms with antibacterial activity.

Scorpion Species	Peptides	Amino Acid Sequence and Length	* Hydrophobicity (kcal × mol^−1^)	* Molecular Weight (Da)	* pI	* Net Charge	Antimicrobial Activity	References
*H. spinifer*	Heterin-1	GVWDWLKKTAKNVWNSDIVKQLKGKAINAAKNYVAEKIGATPS (43)	38.71	4739.5	10.41	+5	Gram-positive and Gram-negative bacteria	[73]
*S. maurus palmatus*	Smp43	GVWDWIKKTAGKIWNSEPVKALKSQALNAAKNFVAEKIGATPS (43)	35.61	4651.5	10.43	+4	Gram-positive and Gram-negative bacteria	[78]
*P. schlechteri*	parabutoporin	FKLGSFLKKAWKSKLAKKLRAKGKEMLKDYAKGLLEGGSEEVPGQ (45)	53.76	4991.8	10.51	+7	Gram-positive and Gram-negative bacteria	[79]
*M. eupeus*	Meucin-49	FKFGSFIKRMWRSKLAKKLRAKGKELLRDYANRVLSPEEEAAAPAPVPA (49)	46.91	5570.1	10.96	+7	Gram-positive and Gram-negative bacteria	[74]
*H. aztecus*	Hadrurin	GILDTIKSIASKVWNSKTVQDLKRKGINWVANKLGVSPQAA (41)	31.56	4433.5	10.87	+5	Gram-negative bacteria	[75]
*P. imperator*	Pandinin-1	GKVWDWIKSAAKKIWSSEPVSQLKGQVLNAAKNYVAEKIGATPT (44)	36.03	4796.6	10.28	+4	Gram-positive bacteria	[72]
*O. carinatus*	Opistoporin 1	GKVWDWIKSTAKKLWNSEPVKELKNTALNAAKNLVAEKIGATPS (44)	41.05	4833.6	10.34	+4	Gram-negative bacteria	[80]
*V. mexicanus*	Vejovine	GIWSSIKNLASKAWNSDIGQSLRNKAAGAINKFVADKIGVTPSQAAS (47)	33.23	4869.5	10.73	+4	Gram-negative bacteria	[76]
*M. martensii*	BmKbpp	FRFGSFLKKVWKSKLAKKLRSKGKQLLKDYANKVLNGPEEEAAAPAE (47)	51.91	5317.9	10.59	+7	Gram-negative bacteria	[77]

* PeP Draw: www.tulane.edu/~biochem/WW/PepDraw/index.html, accessed on 22 October 2024.

**Table 3:** DBPs derived from scorpion venoms with antibacterial activity.

Scorpion Species	Peptides	Peptide Length (S–S Bridge)	Sequence	Classification	Antimicrobial Activity	References
*P. imperator*	Scorpine	75 (3)	GWINEEKIQKKIDERMGNTVLGGMAKAIVHKMAKNEFQCMANMDMLGNCEKHCQTSGEKGYCHGTKCKCGTPLSY	KTx	Gram-positive and Gram-negative bacteria	[82]
*M. martensii*	BmKDfsin4	37 (3)	GFGCPFNQGQCHKHCQSIRRRGGYCDGFLKTRCVCYR	KTx	Gram-negative bacteria	[84]
*L. australasiae*	LaIT2	59 (3)	AKKPFVQRVKNAASKAYNKLKGLAMQSQYGCPIISNMCEDHCRRKKMEGQCDLLDCVCS	KTx	Gram-negative bacteria	[81]
*L. australasiae*	LaIT3	84 (3)	GGILREKYFHKAADALTSNIPIPVVKDVLKSAANQMIRKIGKVQQACAFNKDLAGWCEKSCQEAEGKKGYCHGTKCKCGKPIDY	KTx	Gram-negative bacteria	[85]
*T. discrepans*	Bactridines 1	61 (4)	KDGYIIEHRGCKYSCFFGTNSWCNTECTLKKGSSGYCAWPACWCYGLPDNVKIFDSNNLKC	NaTx	Gram-positive and Gram-negative bacteria	[86]
*T. discrepans*	Bactridines 2	64 (4)	KDGYLVGNDGCKYSCFTRPGTYCANECSRVKGKDGYCYAWMACYCYSMPNWVKTWNRATNRCGR	NaTx	Gram-positive and Gram-negative bacteria	[86]

**Table 4:** NDBPs derived from scorpion venoms with antifungal activity.

Scorpion Species	Peptides	Amino Acid Sequence and Length	* Hydrophobicity (kcal × mol^−1^)	* Molecular Weight (Da)	* pI	* Net Charge	Antimicrobial Activity	References
*T. stigmurus*	Stigmurin	FFSLIPSLVGGLISAFK (17)	3.44	1795.0	9.80	+1	*C. albicans*, *C. krusei*, and *C. glabrata*	[50]
*T. stigmurus*	StigA6	FFSLIPKLVKGLISAFK (17)	7.43	1907.1	10.86	+3	*C. albicans*, *C. krusei*, and *C. glabrata*	[51]
*T. stigmurus*	StigA16	FFKLIPKLVKGLISAFK (17)	9.77	1948.2	11.03	+4	*C. albicans*, *C. krusei*, and *C. glabrata*	[51]
*T. stigmurus*	StigA25	FFSLIPSLVKKLIKAFK (17)	9.08	1978.2	11.03	+4	*C. albicans*, *C. krusei*, and *C. glabrata*	[52]
*T. stigmurus*	StigA31	FFKLIPKLVKKLIKAFK (17)	13.76	2060.3	11.25	+6	*C. albicans*, *C. krusei*, and *C. glabrata*	[52]
*T. obscurus*	ToAP1	FIGMIPGLIGGLISAFK (17)	5.33	1732.9	9.80	+1	*Candida* spp. and *C. neoformans*	[94]
*T. obscurus*	ToAP3	FIGMIPGLIGGLISAIK (17)	5.92	1699.0	9.80	+1	*Candida* spp. and *C. neoformans*	[94]
*A. amoreuxi*	GK-19	GFLFKLIPKAIKKLISKFK (19)	14.71	2218.4	11.25	+6	*C. albicans*, *C. krusei*, and *C. glabrata*	[95]
*T. stigmurus*	TistH	ADMDFTGIAESIIKKIKETNAKPPA (25)	32.02	2687.4	7.07	0	*C. albicans*, *C. tropicalis*, and *A. flavus*	[93]
*T. obscurus*	ToAP2	FFGTLFKLGSKLIPGVMKLFSKKKER (26)	20.81	2998.7	11.22	+6	*Candida* spp. and *C. neoformans*	[94]
*O. cayaporum*	Con10	FWSFLVKAASKILPSLIGGGDDNKSSS (27)	19.82	2823.5	9.59	+1	*Candida* spp. and *C. neoformans*	[94]
*P. imperator*	Pantinin-2	IFGAIWKGISSLL (13)	4.76	1403.8	10.14	+1	*C. tropicalis*	[53]
*P. imperator*	Pantinin-3	FLSTIWNGIKSLL (13)	4.08	1490.8	10.14	+1	*C. tropicalis*	[53]
*P. imperator*	Pantinin-1	GILGKLWEGFKSIV (14)	12.04	1545.9	9.93	+1	*C. tropicalis*	[53]
*T. serrulatus*	TsAP-2	FLGMIPGLIGGLISAFK (17)	5.20	1732.9	9.80	+1	*C. albicans*	[55]
*A. amoreuxi*	AamAP1	FLFSLIPHAIGGLISAFK (18)	5.15	1930.1	9.80	+1	*C. albicans*	[63]
*A. amoreuxi*	AamAP2	FPFSLIPHAIGGLISAIK (18)	7.13	1880.1	9.80	+1	*C. albicans*	[63]
*A. aeneas*	AaeAP1	FLFSLIPSVIAGLVSAIRN (19)	2.78	2016.2	10.60	+1	*C. albicans*	[56]
*A. aeneas*	AaeAP2	FLFSLIPSAIAGLVSAIRN (19)	3.74	1988.1	10.60	+1	*C. albicans*	[56]
*P. imperator*	Pandinin-2	FWGALAKGALKLIPSLFSSFSKKD (24)	15.18	2610.4	10.62	+3	*C. albicans*	[72]
*C. suffuses*	Css54	FFGSLLSLGSKLLPSVFKLFQRKKE (25)	14.79	2868.6	11.02	+4	*C. albicans*	[96]
*T. serrulatus*	Serrulin	GFGGGRGGFGGGRGGFGGGGIGGGGFGGGYGGGKIKG (37)	37.23	3046.5	11.57	+4	*A. niger* and *C. albicans*	[92]
*O. carinatus*	Opistoporin 1	GKVWDWIKSTAKKLWNSEPVKELKNTALNAAKNLVAEKIGATPS (44)	41.05	4833.6	10.34	+4	*N. crassa*, *B. cinerea*, *F. culmorum*, and *S. cervisiae*	[80]
*P. schlechteri*	parabutoporin	FKLGSFLKKAWKSKLAKKLRAKGKEMLKDYAKGLLEGGSEEVPGQ (45)	53.76	4991.8	10.51	+7	*N. crassa*, *B. cinerea*, *F. culmorum*, and *S. cervisiae*	[80]

* PeP Draw: www.tulane.edu/~biochem/WW/PepDraw/index.html, accessed on 22 October 2024.

**Table 5:** DBPs derived from scorpion venoms with antifungal activity.

Scorpion Species	Peptides	Peptide Length (S–S Bridge)	Sequence	Classification	Antimicrobial Activity	References
*T. serrulatus*	Ts8	60 (3)	KLVALIPNDQLRSILKAVVHKVAKTQFGCPAYEGYCNDHCNDIERKDGECHGFKCKCAKD	KTx	*P. pastoris*	[98]
*T. serrulatus*	Ts1	61 (4)	KEGYLMDHEGCKLSCFIRPSGYCGRECGIKKGSSGYCAWPACYCYGLPNWVKVWDRATNKC	NaTx	*A. nidulans*	[97]

## Abbreviations

AMPs	antimicrobial peptides
AMR	antimicrobial resistance
DBPs	disulfide-bridged peptides
CaTx	calcium channel toxins
ClTx	chloride channel bound toxins
HIV	human immunodeficiency virus
IC_50_	50% inhibitory concentration
LPS	lipopolysaccharides
LTA	lipoteichoic acid
MIC	minimum inhibitory concentration
MRSA	methicillin-resistant Staphylococcus aureus
KTx	potassium channel toxins
NaTx	sodium channel toxins
NDBPs	non-disulfide-bridged peptides
TRPTx	TRP channel toxins
µM	micromolar
